# 

**Published:** 1888-10

**Authors:** 


					﻿Vol. VIIL
* OCTOBER, 1888.
NO. la
THE
HOMEOPATHIC pHY^ffilAfl,
A Monthly Journal of Homoeopathic Materia Medica
and Clinical Medicine.
“ He is a freeman whom truth makes free, and all are slaves beside"
"Seek the Truth: come whence it may, cost what it will."
EDITED AND PUBLISHED BY
fexMUNIi J. LEE, Ki.
AND
WALTER M. JAMES, E>.
PHILADELPHIA:
No. 1133 SPRUCE STREET.
O "NT X> O 3ST:
ALFRED HEATH & CO., Sole Agents fob Great Britain,
114 Ebury Street, Eaton Square.
MS^Tearly Subscription, $2.50. invariably in advance. Single numbers, 25 cts.
Entered at the Philadelphia Poet-Office as Second- Class Hall Matter.
				

